# Splenic infarction secondary to polycythemia Vera: Case report and literature review

**DOI:** 10.1016/j.radcr.2023.07.049

**Published:** 2023-08-04

**Authors:** Qusay Abdoh, Mohammad Alnees, Islam Rajab, Alaa Zayed, Hamza Salim, Abdelkarim Barqawi, Riad Amer

**Affiliations:** a Department of Medicine, Faculty of Medicine & Health Sciences, An-Najah National University, Nablus, Palestine; b Department of Internal Medicine, GI and Endoscopy unit, An-Najah National University Hospital, Nablus, Palestine

**Keywords:** Splenic, Infarction, Secondary, Polycythemia Vera, Case, Report

## Abstract

Splenic infarction is a medical condition characterized by compromised blood flow to the spleen, resulting in partial or complete organ infarction. This condition is commonly observed in patients with an increased risk of thrombosis, such as those with Polycythemia Vera (PV).

A 40-year-old female patient presented with fatigue, weakness, and an enlarged spleen, further tests revealed elevated levels of hemoglobin, white blood cells, and platelets. A bone marrow biopsy and positive Jack II mutations confirmed the diagnosis of PV. The patient later developed portal hypertension, varices, and splenic infarction.

This case report aims to raise awareness about the potential complications of PV and emphasizes the importance of early intervention to prevent serious consequences such as splenic infarction. Additionally, it highlights the role of splenectomy in managing complications associated with PV.

## Introduction

Polycythemia Vera (PV) is a condition where the body produces too many red blood cells (RBC), leading to an elevated RBC mass. This can also result in increased production of white blood cells and platelets. The cause of this disorder is believed to be due to abnormal hematopoietic cell clones, which make the body more sensitive to growth factors. Symptoms such as headaches, dizziness, and thrombosis are caused by the increased viscosity of the blood [1].

PV is associated with various complications such as thrombosis, hemorrhage, peptic ulcer disease, myelofibrosis, acute leukemia, or myelodysplastic syndrome (MDS) [1]. Thrombosis is a significant concern in PV, and the current treatment strategies focus on reducing the risk of thrombosis [2].

Individuals diagnosed with myeloproliferative neoplasms are at a significantly higher risk of experiencing thrombotic events. Several studies have identified the presence of an underlying myeloproliferative neoplasm in patients who developed portal hypertension with esophagus and/or fundal varices [3].

This report presents a case of PV with portal hypertension (PHT) and perisplenic, perigastric varices complicated by splenic infarction. The significance of recognizing splenic infarction as a potential complication in patients with PV is emphasized. It is crucial to promptly investigate for complications once the hematological diagnosis is confirmed. Furthermore, the study highlights the role of splenectomy in managing complications associated with PV.

## Case presentation

A 40-year-old female patient presented with 4 months duration of fatigue and general weakness. On further investigations high hemoglobin levels (>16.5 g/dL), high white blood cell count, and platelets, along with splenomegaly. She was diagnosed with PV based on a bone marrow biopsy and positive Jack II mutations. The patient underwent therapeutic phlebotomy until her hemoglobin levels dropped to 11.5 g/dL, but her condition worsened during follow-up. She complained of severe exertional dyspnea, fatigue, and weakness. Complete blood count (CBC) revealed hemoglobin levels of 5.6 g/dL, and further investigations showed iron deficiency anemia (IDA). The patient was started on intravenous (IV) iron once weekly but her hemoglobin levels dropped to 4.0 g/dL.

The patient was admitted for blood transfusion and further investigation to rule out splenic vein thrombosis. On admission, the patient's vital signs were stable, and a physical examination revealed palpable splenomegaly. The patient had no relevant family history of medical conditions or genetic predispositions, and her psychosocial history revealed that she is married, has 2 children, and works as a teacher with no history of substance abuse.

Laboratory findings revealed slightly elevated INR values, while GGT, ALT, AST, ALP, TSB, and albumin levels were normal. Potassium levels were at the lower borderline. The PT, PTT, and fibrinogen levels were normal.

**Fig. 1:**
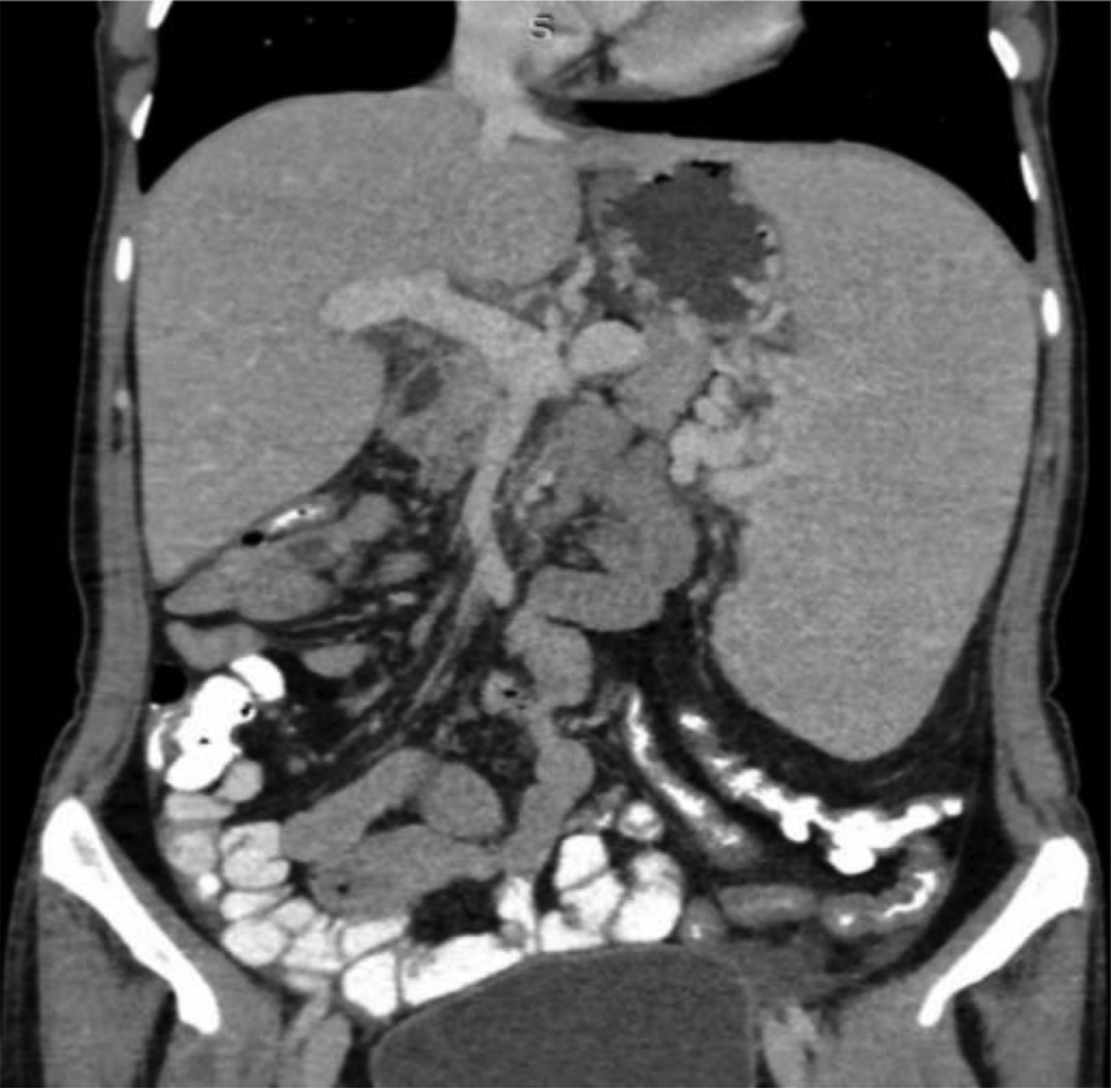
A CT scan with IV contrast revealed an enlarged spleen with peri-gastric and splenic varices, as well as dilated portal and splenic veins. There is no evidence of nodules, irregularities, or any signs of liver cirrhosis.

A CT scan with IV contrast revealed an enlarged spleen with peri-gastric and splenic varices, dilated portal and splenic veins, and no evidence of filling defect, ruling out splenic vein thrombosis (“Fig. 1”).

**Fig. 2:**
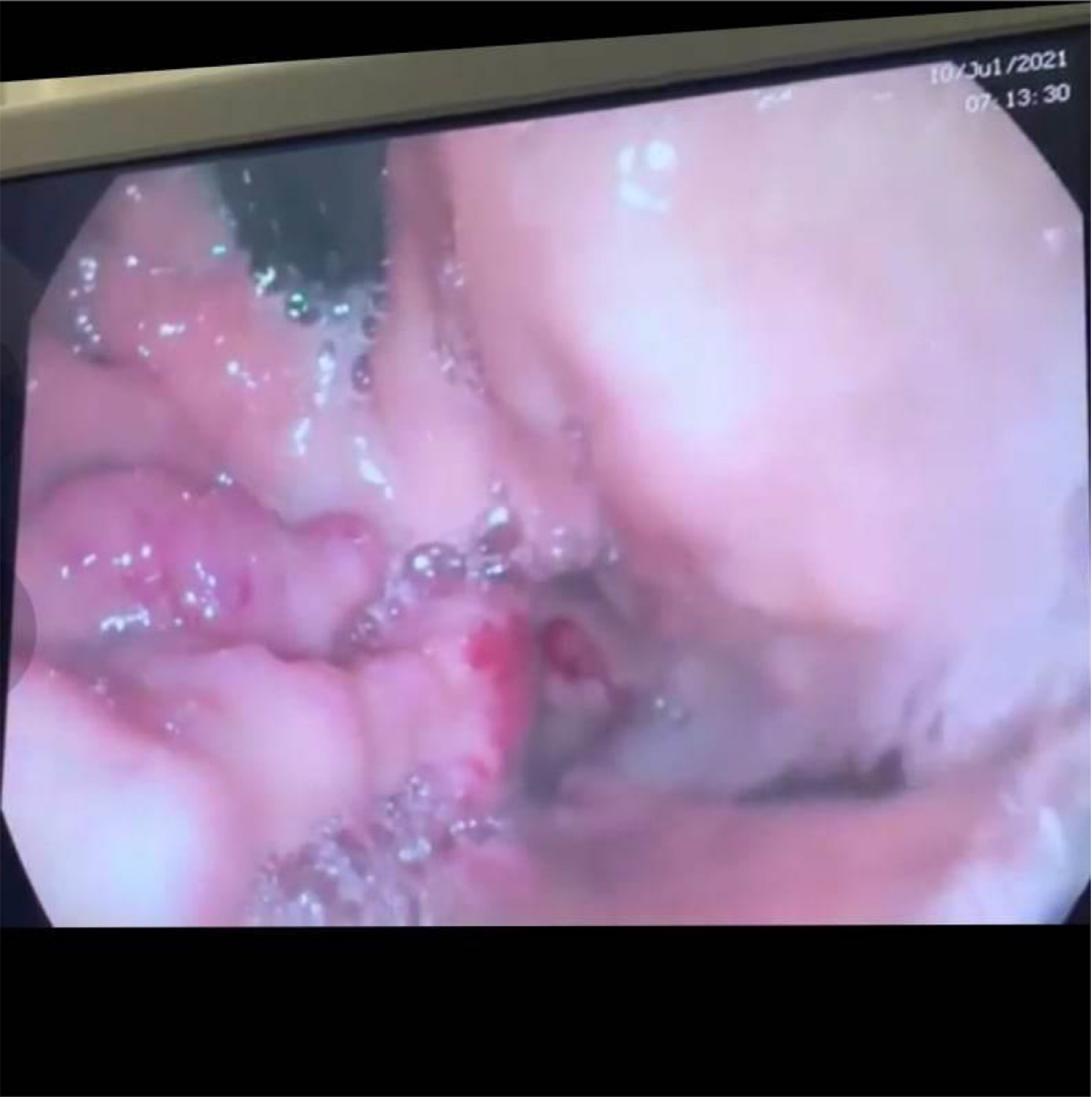
Upper GI endoscopy, revealing multiple small tortuous superficial fundal varices with stigmata of recent bleeding.

Due to concern about bleeding, the patient underwent an upper GI endoscopy, revealing multiple small tortuous superficial fundal varices with stigmata of recent bleeding (“Fig. 2”).

**Fig. 3:**
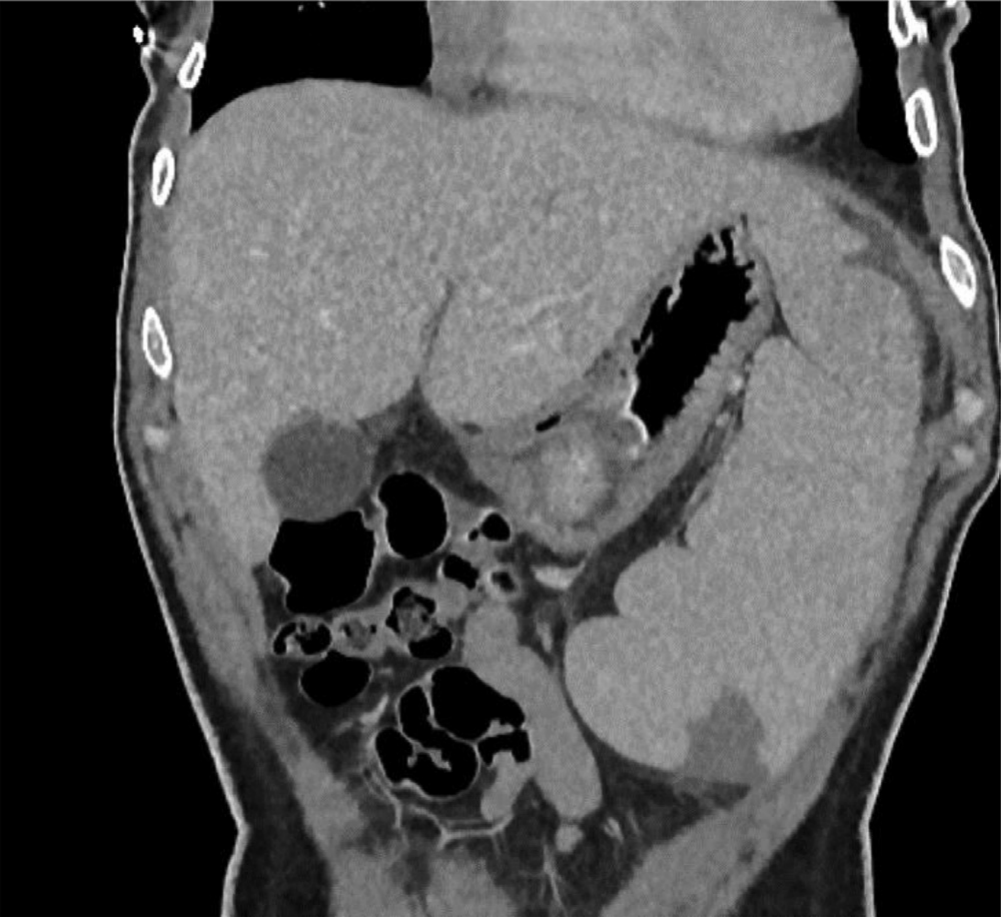
Abdomen CT scan revealed wedge-shaped splenic infarction.

During admission, the patient experienced severe abdominal pain in the left upper quadrant, and a subsequent abdomen CT scan revealed 2 wedge-shaped splenic infarctions. A multidisciplinary team agreed to perform a splenectomy (“Fig. 3”).

**Fig. 4:**
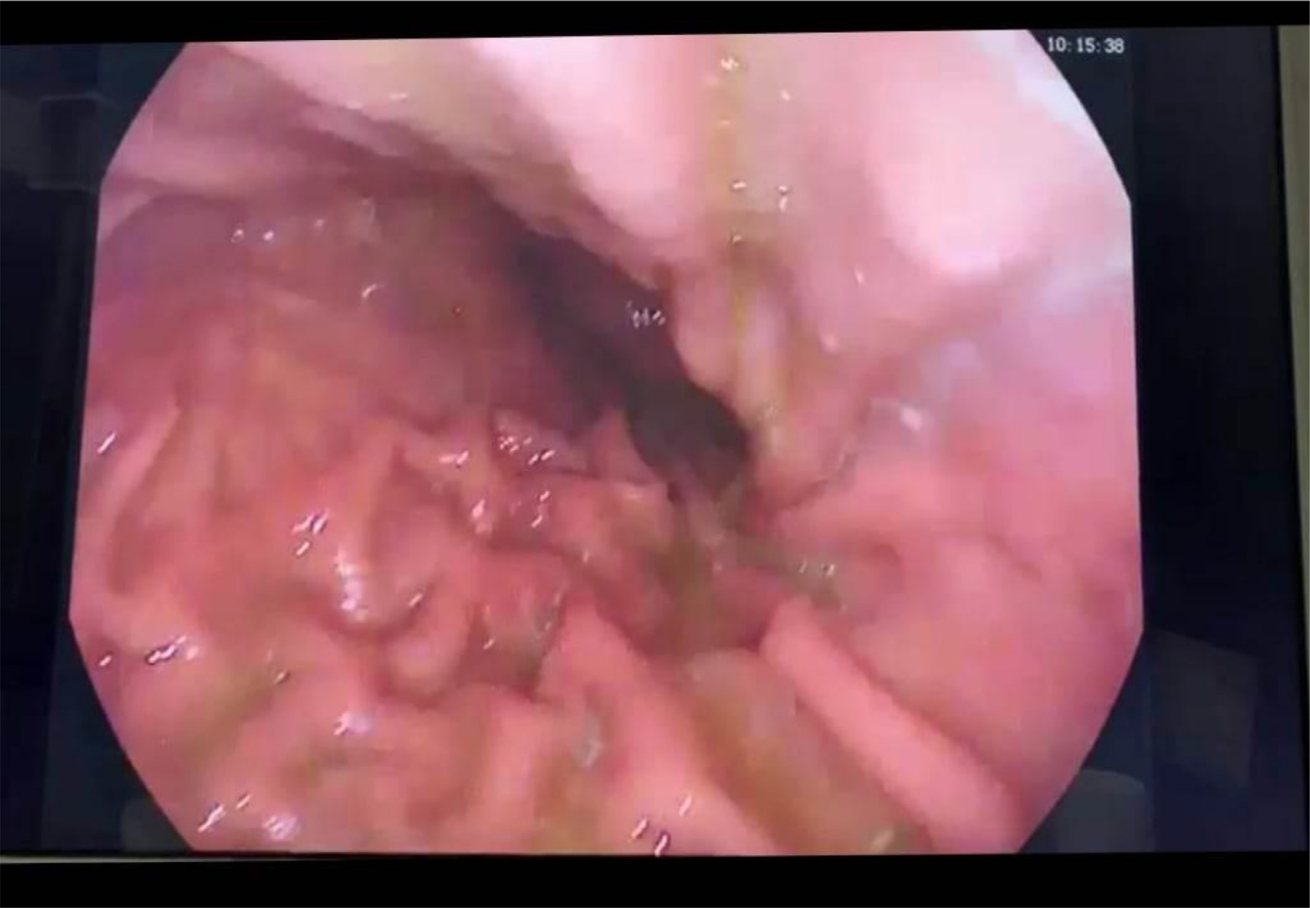
Follow-up endoscopy showed complete resolution of gastric varices.

**Fig. 5:**
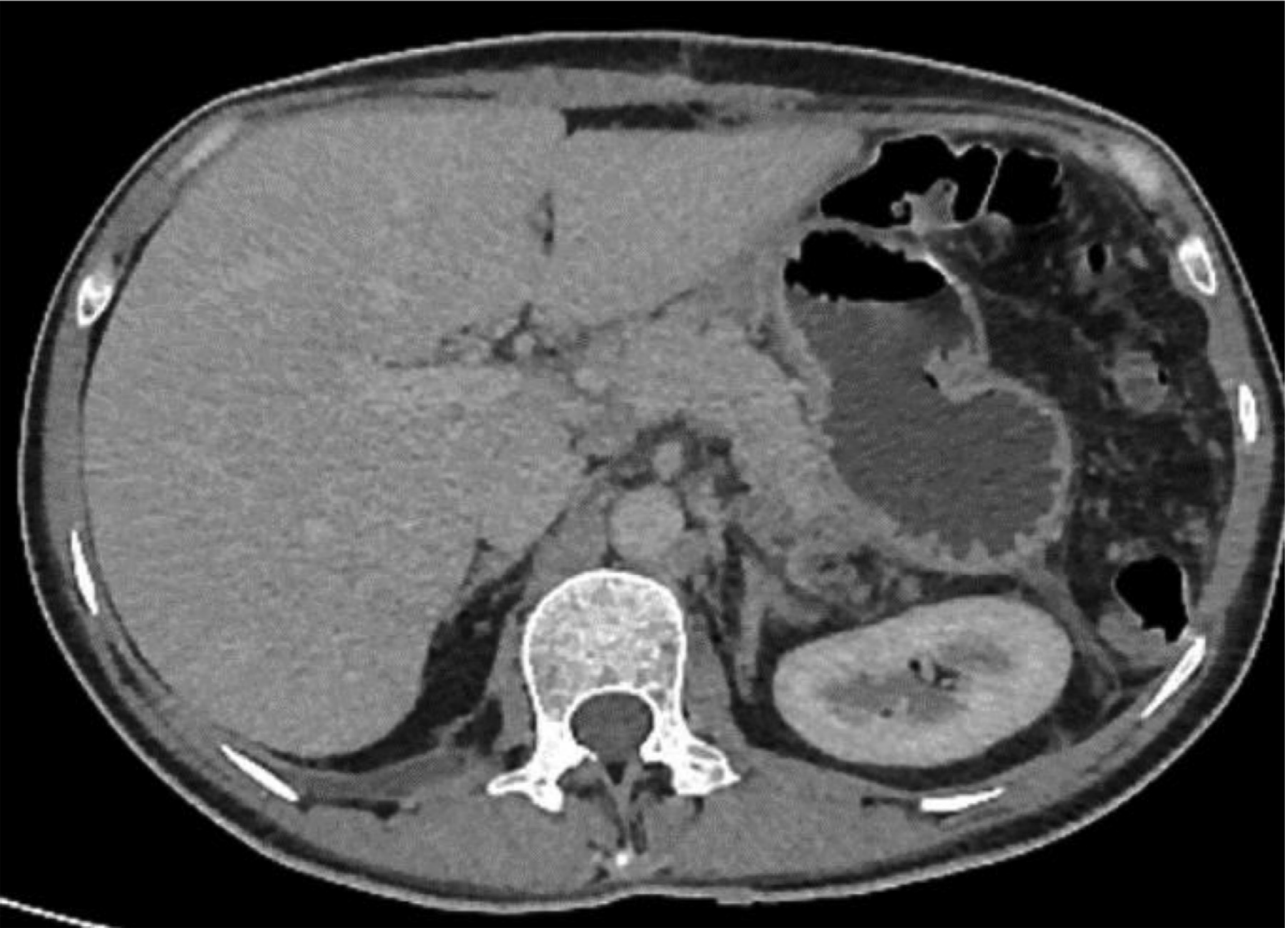
A follow-up abdomen CT scan conducted revealed resolution of perigastric and perisplenic varices.

The procedure was completed without complications, and follow-up endoscopy showed complete resolution of gastric varices (“Fig. 4”). The patient was maintained on anticoagulant therapy and IV iron. The most recent CBC showed an increase in hemoglobin levels from 4.0 g/dL to 14.9 g/dL. A follow-up abdomen CT scan conducted revealed resolution of perigastric and perisplenic varices (“Fig. 5”).

## Discussion

Polycythemia, also known as erythrocytosis, is marked by an increase in RBC mass. In practice, this manifests as a rise in hemoglobin concentrations, or hematocrit, above what is deemed normal for the age and gender [4]. Polycythemia could be either primary or secondary. Primary polycythemia is caused by neoplastic processes, such as the uncontrolled proliferation of hematopoietic cells in primary familial polycythemia and myeloproliferative diseases. Secondary polycythemia is induced by an increase in erythropoietin production resulting from a variety of factors, including cancer, persistent hypoxia, and hemoglobinopathies [5].

PV is an acquired, Philadelphia-chromosome-negative myeloproliferative disorder linked in 90% of cases with Janus kinase-2 (JAK2) gene mutations that result in signaling abnormalities. An abnormal response to growth factors and constitutive activation of cytokine receptors arise from a JAK2V617F mutation, where valine is replaced by phenylalanine at codon 617 [1]. Due to uncontrolled neoplastic proliferation, the prepolycythemic and overt polycythemic bone marrow (BM) findings are important for hypercellularity and panmyelosis, with a varied proliferation of granulocyte precursors, erythrocytes, and megakaryocytes, with the erythrocyte being the most overproduced [5] (Table 1).

**Table 1:** The patient's labs on admission.

Parameter	Value	Reference ranges
Aminotransferase, alanine (ALT)	27.4	4-36 U/L
Aminotransferase, aspartate (AST)	30.5	8-33 U/L
Glutamyl transpeptidase, Gamma (GGT)	24	5-36 U/L
Alkaline phosphatase (ALP)	57	30-120 U/L
Hemoglobin	8.45	12-16 g/dL
White blood count	3.62	4.5-11.0 × 10^9^/L
Platelet count	159.2	150-400 × 10^9^/L
Prothrombin time (PT)	15.9	11-13 s
Activated partial thromboplastin time	28.8	25-35 s
Fibrinogen	245	200-400 mg/dL
INR	1.23	˂1.1
Albumin	4.44	3.5-5.5 g/dL
Total serum bilirubin (TSB)	0.6	0.3-1.0 mg/dL
Blood urea nitrogen (BUN)	8.6	8-20 mg/dL
Creatine	0.45	0.50-1.10 mg/dL
Na	142	136-145 mEq/L
K	3.29	3.5-5.0 mEq/L
Cl	109.2	98-106 mEq/L

The most common symptoms reported by PV patients are headache, fatigue, dizziness, paresthesia erythromelalgia, and visual disturbances [6]. These symptoms are caused by reduced capillary blood flow, microvascular thrombi, and fundoscopic signs of hemorrhage and clogged veins. Forty percent of patients have aquagenic pruritus, which manifests itself during or after a hot shower and is attributed to a histamine surge that comes from mast cell and basophil degranulation [1]. Some patients may also present with high blood cell counts identified inadvertently during evaluations for other conditions. On physical exam, splenomegaly, hepatomegaly, and hypertension may be detected [7]. The cause of these signs and symptoms is a hyperviscosity condition brought on by an excess of erythropoiesis [8].

PV has been described as a rare complication with an unknown incidence of splenic infarction by several causes, the most prevalent of which is a thromboembolic disorder. Thromboembolic events typically happen right before or right after diagnosis and become less frequent over time, most likely as a result of the effects of therapy [9].

Splenic infarction results when the spleen's arterial blood supply is compromised, causing tissue ischemia and necrosis. Blood-borne malignancies, myelofibrosis, hypercoagulable states, thromboembolic diseases, and blunt abdominal injuries are some of the possible causes of splenic infarction [10].

Both microvascular and macrovascular thrombotic problems occur in PV. While macrovascular complications are brought on by thrombus formation in large blood vessels, microvascular complications, such as headaches, visual disturbances, tinnitus, and so on, are brought on by thrombus formation in small blood vessels [11].

The pathophysiology of thrombosis is multifactorial and includes several factors, including blood cells, endothelial cells, the coagulation system, and inflammatory mediators, hemostatic components, cytokine dysregulation, and endothelial dysfunction all contribute to thrombogenesis. Furthermore, a hyperviscosity state due to excess erythropoiesis leads to a hypercoagulable state and thrombotic events [8].

Despite presenting with severe anemia, with a hemoglobin level of 4, our patient experienced splenic infarction. This prompted us to investigate additional mechanisms of thrombosis in PV, beyond the commonly known hyperviscosity or erythrocyte-related factors that contribute to hyperviscosity.

The leading cause of mortality and morbidity in PV is thrombotic events, which involve both arterial and venous events. Myocardial infarction, ischemic stroke, and peripheral artery occlusion are twice as common as venous thrombosis. Pulmonary embolism, Budd Chiari syndrome, deep vein thrombosis, and, less often, cerebral venous sinus thrombosis and splanchnic venous thrombosis are some of the venous events that might occur in PV patients [8].

Patients with PV have an increased risk of developing blood clots in both veins and arteries. The incidence of these events is estimated to be between 0.4 and 2.8 cases per 100,000 individuals per year, and the lifetime prevalence is around 20%-30% according to an international study [12,13]. PV is a common risk factor for noncirrhotic portal vein thrombosis [14]. Portal vein thrombosis can transform liver tissue, resulting in the formation of collateral circulation, ascites, portal hypertension, biliary disease, esophagogastric varices, and gastrointestinal bleeding [15].

Portal hypertension is an abnormal condition that affects blood flow in the liver and can cause serious complications such as ascites, variceal hemorrhage, hepatic encephalopathy, and cirrhosis. Portal hypertension can be categorized as either cirrhotic or noncirrhotic [15]. Noncirrhotic portal hypertension (NCPH) is a group of liver conditions that mainly impact the liver's vascular system. NCPH etiology is categorized based on the location of blood flow resistance, including prehepatic, hepatic, and posthepatic [16].

PV one of the causes that contribute to the development of noncirrhotic portal hypertension. When PV occurs alongside portal hypertension, it is categorized as prehepatic NCPH [15]. In our case, NCPH was developed without evidence of portal or splenic vein thrombosis.

The best diagnostic method for splenic infarction is CT (computerized tomography) scanning with contrast and appears as a wedge-shaped, sharply contoured hypodense lesion in CT scanning [17].

As there is currently no cure for PV, therapy focuses on relieving symptoms and preventing complications such as bleeding, thrombosis, and the progression of myelofibrosis, acute leukemia, or myelodysplastic syndrome. Phlebotomy, aspirin, and lifestyle changes are used to treat patients younger than 60 who have no history of thrombotic episodes, whereas cytoreductive treatment is used for patients older than 60 and/or those with a thrombotic history. In 2014, the Food and Drug Administration authorized a JAK inhibitor (ruxolitinib) for the treatment of individuals with PV who had an unsatisfactory response to hydroxyurea or were intolerant of it. Complete hematological remission was obtained in a substantial majority of ruxolitinib-treated individuals [18].

Splenectomy may be appropriate in the setting of symptomatic portal hypertension (eg, variceal bleeding, ascites), painful splenomegaly, recurrent splenic infarcts, or established RBC transfusion-dependent anemia [1,18].

The patient underwent a successful splenectomy procedure, which resulted in complete resolution of gastric varices as confirmed by follow-up endoscopy and abdomen CT scan. The most recent CBC revealed a significant increase in hemoglobin levels, indicating the effectiveness of the intervention. The resolution of perigastric and perisplenic varices further highlights the success of the splenectomy. Overall, these findings demonstrate that splenectomy is an effective intervention for PV patients with symptomatic portal hypertension (eg, variceal bleeding, ascites) and splenic infarctions.

## Patient consent

Written informed consent for the publication of this case report was obtained from the patient.
